# Enhanced Oxygen‐Reaction Electrocatalysis and Corrosion Resistance of CoCrFeNi Thin Films by Tuned Microstructure and Surface Oxidation

**DOI:** 10.1002/smsc.202400296

**Published:** 2024-09-29

**Authors:** Clara Linder, Robert Boyd, Grzegorz Greczynski, Mikhail Vagin, Daniel Lundin, Karin Törne, Per Eklund, Emma M. Björk

**Affiliations:** ^1^ Division Materials and Production, Corrosion RISE Research Institutes of Sweden 164 40 Kista Sweden; ^2^ Nanostructured Materials Department of Physics, Chemistry and Biology (IFM) Linköping University 581 83 Linköping Sweden; ^3^ Thin Film Physics Division Department of Physics, Chemistry, and Biology (IFM) Linköping University 581 83 Linköping Sweden; ^4^ Laboratory of Organic Electronics Department of Science and Technology Linköping University 601 74 Norrköping Sweden; ^5^ Plasma and Coatings Physics Division Department of Physics, Chemistry, and Biology (IFM) Linköping University 581 83 Linköping Sweden; ^6^ Department of Chemistry—Ångström Uppsala University 751 21 Uppsala Sweden

**Keywords:** anodization, corrosion, high-power impulse magnetron sputtering, magnetron sputtering, oxygen electrocatalysts

## Abstract

Oxygen electrocatalysts play a key role in renewable and fossil‐free energy production. Bifunctional catalysts active for oxygen reduction reaction (ORR) and oxygen evolution reaction (OER) allow use of the same material system for both energy production (ORR) and fuel generation (OER). However, optimizing the performance of bifunctional catalysts requires in depth understanding of the catalyst structure, its surface chemistry in terms of active sites and the underlying catalytic mechanism. Here, the catalytic performance of CoCrFeNi thin films is investigated, synthesized using high‐power impulse magnetron sputtering, as bifunctional oxygen electrocatalysts. The film crystal structure and morphology, and thereby the catalytic performance, can be tuned by the ion acceleration (bias) to the substrate. To further enhance the catalytic activity, anodization is used to electrochemically modify the films, forming a thicker oxide layer enriched in Co and Ni cations which significantly improves the ORR performance. Anodization improves the catalyst stability during OER, with an OER potential of 1.45 V versus the reversible hydrogen electrode (RHE) at 10 mA cm^−2^ for more than 24 h. While the corrosion resistance is high both before and after anodization, in terms of catalytic activity the anodized films outperformed the as‐deposited ones. This makes anodized films excellent electrocatalyst candidates in corrosive alkaline environments such as fuel cells and electrolyzers.

## Graphical abstract

This work presents CoCrFeNi thin films, synthesized by physical vapor deposition as bifunctional oxygen electrocatalysts that are corrosion resistant in alkaline conditions. The film microstructure is tuned by ion acceleration (substrate bias) during deposition and the film's surfaces are oxidized electrochemically by anodization. As‐deposited, the films are active OER catalysts and anodization activates the films towards ORR.

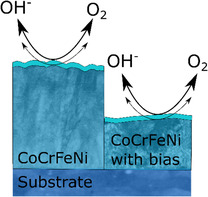

## 1 Introduction

Alkaline fuel cells and anion exchange membrane (AEM) fuel cells are low‐carbon‐footprint production methods for electricity.^[^ ^1^, ^2^, ^3^ ^]^ These technologies rely on electrochemical hydrogen oxidation reaction (HOR) and oxygen reduction reaction (ORR) that take place at the active membranes or electrodes.

(1) $$\text{HOR}:{\text{ H}}_{2}+2{\text{OH}}^{-}↔2H_{2}O+2e^{-}$$

(2) $$\text{ORR}:O_{2}+2H_{2}O+4e^{-}↔4{\text{OH}}^{-}$$

ORR is typically the limiting reaction of the process because of its sluggish kinetics and the corresponding need for an electrocatalyst. Pt‐based catalysts are very common due to their high catalytic activity.^[^ ^2^, ^4^ ^]^ However, cheaper and more abundant catalysts such as oxides of Ni, Co, Fe, and Mn^[^ ^3^, ^4^, ^5^ ^]^ are used today in fuel cells, despite their lower performance. Thus, there is a need to improve these new catalysts so that their performance can match that of the noble metals. One of the most promising candidates is cobalt oxide Co_3_O_4_ for ORR,^[^ ^6^, ^7^, ^8^ ^]^ Other candidates are NiO and Fe_2_O_3_.^[^ ^9^ ^]^

Electrical energy can be stored as hydrogen gas, formed through water electrolysis, and be used as fuel. In electrolysis, water is split into oxygen and hydrogen gas by oxygen evolution reactions (OER) and hydrogen evolution reactions (HER), the reverse reactions to ORR and HOR. Similarly to fuel cells, OER is usually the limiting reaction in the electrolysis process. Thus, there is a need for a catalyst. Noble catalysts and 3*d* transition metals, or combinations of the two, are existing catalysts for OER. Furthermore, some 3*d* transition metal oxide catalysts can be active for both ORR and OER. Co_3_O_4_, NiO, Fe_2_O_3_, NiCo_2_O_4_, CoFe_2_O_4_, and MnFe_2_O_4_^[^ ^9^, ^10^, ^11^, ^12^ ^]^ are thus classified as bifunctional electrocatalysts.

The environment inside alkaline and AEM fuel cells and electrolyzers is highly basic, with high pH of the KOH or NaOH electrolyte.^[^ ^13^ ^]^ This is not an issue for Pt‐based catalysts but challenging for non‐noble metal‐based oxides. Indeed, if the catalysts are not corrosion resistant, they may be dissolved into the electrolyte, poisoning the electrodes, and thus reducing the lifespan of the device. Any new catalyst materials must therefore be able to form protective passive films as physical and chemical barriers against the corrosive environment. Thin films, a few hundred of nanometers thick coatings, can be used to coat surfaces that need to be protected against the corrosive environment. Among physical vapor deposition (PVD) techniques for thin‐film deposition, high‐power impulse magnetron sputtering (HiPIMS) stands out for its ability to ionize the sputtered species which can then be attracted by tuning the substrate bias and deposit highly dense films on complex structures.^[^ ^14^, ^15^, ^16^ ^]^ Another advantage of PVD techniques is the possibility to synthesize nanostructured films with small grain sizes (<10 nm), which can be tailored using substrate bias and HiPIMS.^[^ ^17^ ^]^ Small grain size or grain refinement is known to improve the corrosion resistance of stainless steels^[^ ^18^, ^19^ ^]^ and other metals.^[^ ^20^ ^]^ The higher corrosion resistance of refined materials in acidic and salt environments is attributed to the fast formation of a homogenous passive layer, as more grain boundaries are available for metal diffusion in the early oxidation stages and the formation of the protective passive layer.^[^ ^21^ ^]^

High entropy alloys (HEAs) are multicomponent systems with nearly equimolar compositions, first reported by Yeh et al. in 2004.^[^ ^22^ ^]^ For 3*d* transition metals HEA, the Cantor alloy CoCrFeNiMn is the alloy of highest interest for its mechanical strength and corrosion resistance that exceed conventional alloys.^[^ ^23^, ^24^, ^25^, ^26^, ^27^ ^]^ The HEA CoCrFeMnNi and its variants are also reported as active catalyst toward ORR and OER as nanoparticles.^[^ ^28^, ^29^ ^]^ However, CoCrFeNi as oxygen electrocatalyst synthesized as thin films are yet to be studied.

In this study, we report the effect of direct current (DC) substrate bias for HiPIMS depositions on the synthesis of CoCrFeNi thin films. We investigate the effect of crystal structure, grain size, and defect density on the catalytic activity as well as the corrosion resistance in alkaline environments. To induce additional active sites for catalysis, we used anodization as a surface modification technique, and investigate how it affects the ORR and OER performance compared to the as‐deposited films. The surface chemistry and activity over time of the films is also studied.

## 2 Results

### 2.1 Thin Film Structure and Composition

The chemical composition of the CoCrFeNi thin films deposited on polished steel substrates was determined using scanning electron microscopy and energy dispersive spectroscopy (SEM‐EDS) and is shown in **Table** **1**.

**Table 1:** Compositions of films in at% determined by EDS.

Substrate bias	Cr	Fe	Co	Ni
Floating	22.5 ± 0.4	22.3 ± 0.3	30.1 ± 0.3	25.1 ± 0.2
−100 V	20.8 ± 0.5	19.2 ± 0.6	33.0 ± 0.5	26.9 ± 0.5
−200 V	20.9 ± 0.1	20.1 ± 0.9	35.3 ± 0.1	23.7 ± 0.9
−300 V	23.1 ± 0.5	16.1 ± 0.1	38.4 ± 0.5	22.4 ± 0.4
−400 V	29.9 ± 0.4	13.3 ± 0.3	35.9 ± 0.1	20.9 ± 0.2

The substrate temperature, voltage, pulse length, and pulse frequency were the same for all films, only the substrate bias was varied. As can be seen in Table 1, the Co and Cr content increase with substrate bias, and the Ni and Fe content decrease with substrate bias.

**Figure 1:**
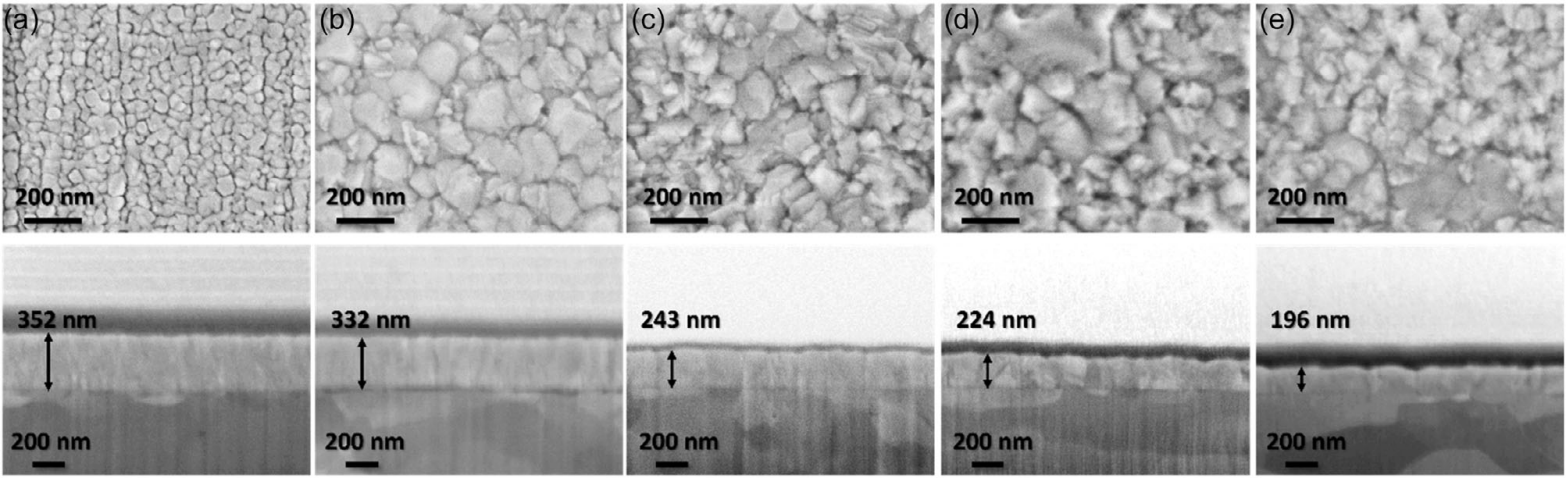
SEM surface (top row) and cross‐section (bottom row) images of the films: a) Floating, b) −100 V and c) −200 V d) −300 V, and e) −400 V substrate bias.

SEM images of the surface morphology and cross‐sections of the films are shown in **Figure** **1**.

The film deposited at floating potential, exhibits regular circular grains of 64 (±8) nm in diameter. For films deposited at −100 V DC substrate bias, the grain size is 148 (±14) nm in diameter, with smaller irregular grains present in between the larger grains. The grain size of the films deposited at higher voltage values are 140 (±11), 144 (±15), and 144 (±12) for the −200, −300, and −400 V film, respectively, similar to the −100 V film but irregular‐sized faceted grains are more common. In the cross‐section images, it can be seen that the column width increases with increasing bias. The film thickness decreases with increasing substrate bias, from 352 to 196 nm.

**Figure 2:**
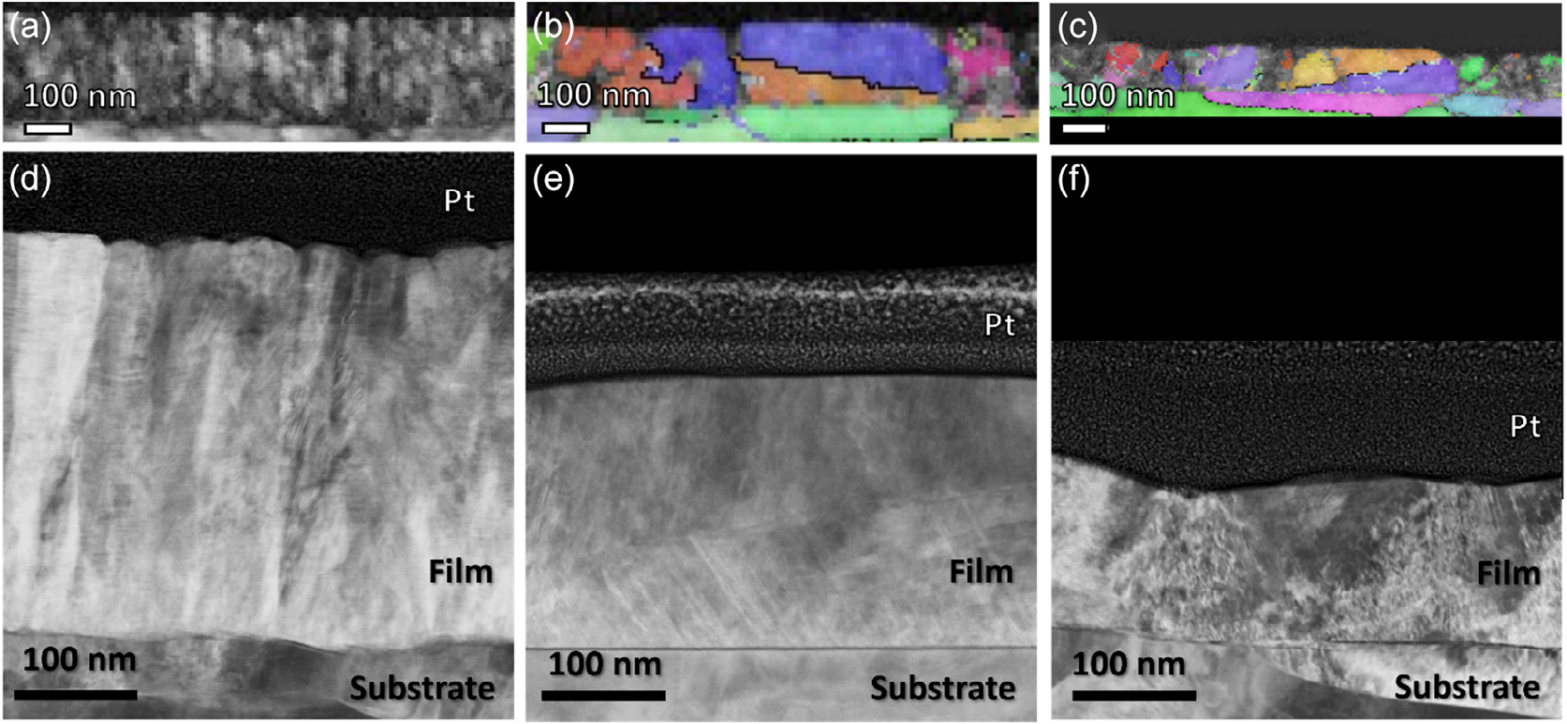
TKD grain maps with inverse pole figure (IPF) superimposed a–c) and dark field STEM images d–f) of the floating (a,d), −200 V (b,e) and −400 V (c,f) film.

To further investigate the changes in microstructure, scanning transmission electron spectroscopy (STEM) analysis was carried out on the films deposited at floating potential and −200 and −400 V bias, shown in **Figure** **2**. The grain structure of the films is most clearly resolved in the band contrast images as determined by the transmission Kikuchi diffraction (TKD) analysis. The film deposited at floating potential has the characteristic structure typical of thin films with column width of ≈45 nm. For films grown with a substrate bias the structure changes. For the −200 V film larger grains are seen (≈220 nm) organized in a random crystal orientation. For the −400 V film, both large and small crystallite domains were found. However, those crystallites are not columnar but equiaxed. Kikuchi patterns obtained from the films could be indexed and detected grains are superimposed on the band contrast images in Figure 2a–c. For the floating film the indexing was complicated because of the small grain size. The larger grains observed for the films deposited at −200 and −400 V potential could be indexed to both face‐centered cubic (FCC) and hexagonal close packed (HCP) structures. The FCC grains tended to be larger compared to the HCP ones, and a higher concentration of HCP grains was observed for the film deposited with a −400 V substrate bias. Dark field STEM image show that the floating film has defects attributed to stacking faults and underdense grain boundaries with intercolumnar voids (shown in the Figure S2, Supporting Information). These defects can be seen in the −200 V film in the grain closest to the substrate interface, but not in the grain domain on top of that. EDS mapping of the film was carried out and is shown in the Figure S3, Supporting Information. The composition of the floating and −400 V film is homogenous over the film thickness, whereas the −200 V film showed Cr‐enriched and Ni‐depleted areas throughout the film thickness. Ar was detected at the substrate film interface for the −400 V film which is a result of ion bombardment and Ar incorporation.

**Figure 3:**
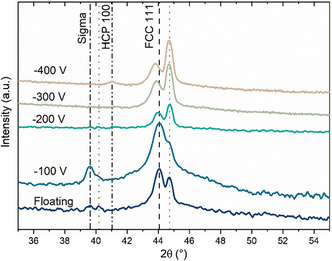
XRD diffractograms for the films obtained with Cu Kα radiation in a 2*θ*/*θ* configuration. Dotted lines are diffractions from the steel substrate.

**Figure** **3** shows the X‐ray diffraction (XRD) diffractograms from the films. The dotted lines correspond to the steel substrate. The films all exhibit an FCC structure with a (111) preferred orientation as seen from the peak at 44°. The position of the peak shifts toward lower angles values with increased bias, indicating a higher stress level within the films. For the floating and −100 V films an additional phase is seen around 40° which is identified as a secondary tetragonal sigma phase. For −400 V film an additional HCP phase with a 100 orientation is seen around 41°.

### 2.2 Electrochemical Activation of Films

**Figure 4:**
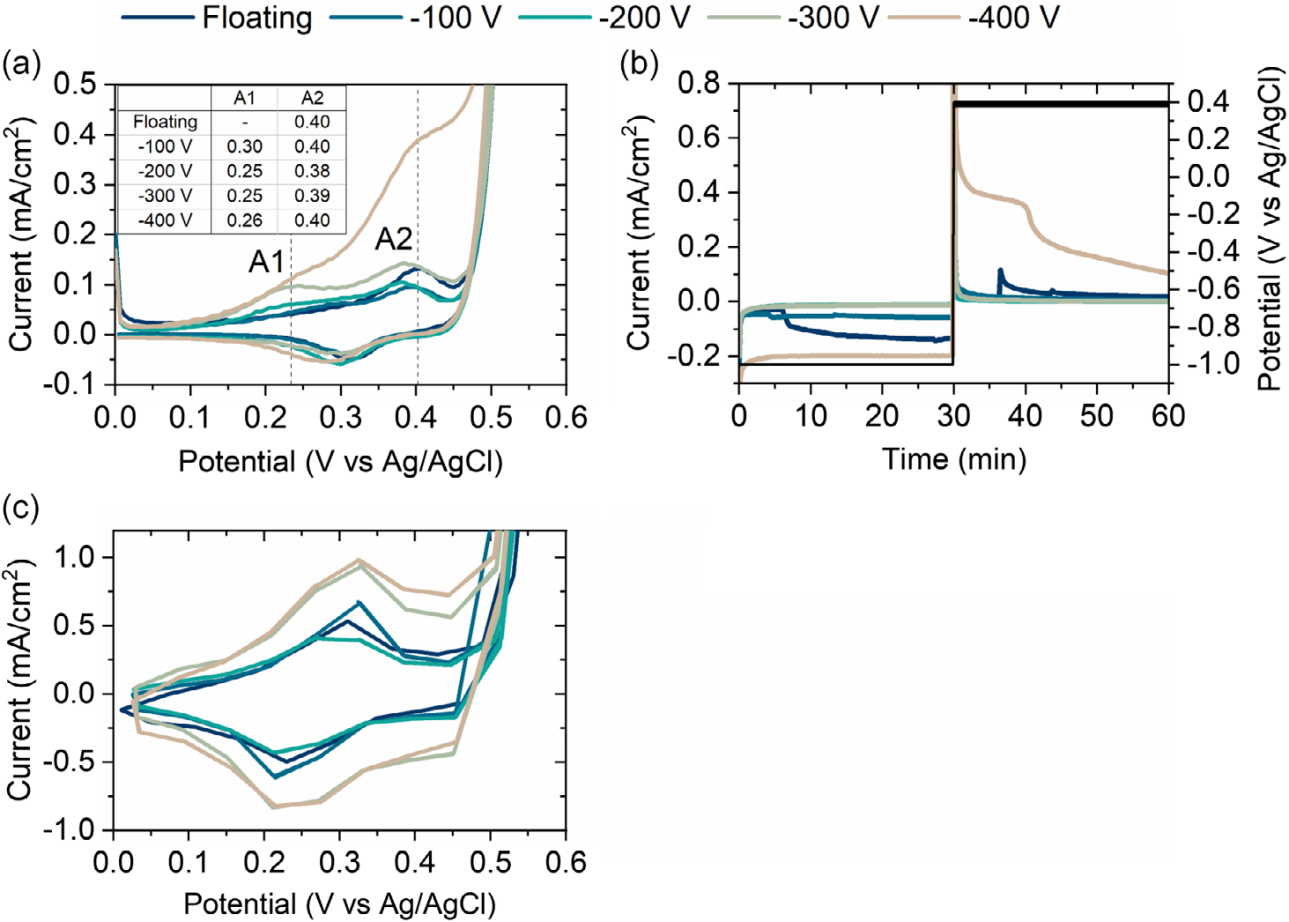
a) CVs of reduced films (after −1 V 30 min), b) anodization profile, and c) final CV in activation step.

As mentioned in the introduction, it is the transition metal oxides that are active toward ORR and OER. Anodization is a common electrochemical technique used to remove the initial surface and construct a new layer with the desired surface chemistry and structure. In our previous study,^[^ ^30^ ^]^ we saw an improvement of the ORR catalytic activity for CoCrFeNi thin films deposited by DCMS after anodization (5 min at −1 V vs Ag/AgCl, then 30 min at 0.32 V vs Ag/AgCl). The native oxide of the films is believed to be reduced back to the metallic states of Co, Cr, Fe, and Ni by applying −1 V for 30 min. The anodization potential to be used in the oxidation step was determined from the cyclic voltammograms of reduced samples, shown in **Figure** **4**a.

In Figure 4a, there is only one visible peak (A2) for the floating film and two peaks (A1–A2) for the other films. The first peak, A1 is associated with the redox couple: Co^2+^/Co^3+^ and the second peak, A2, with: Co^3+^/Co^4+^ and Ni^2+^/Ni^3+^, based on reports for Co_3_O_4_ and NiCo_2_O_4_.^[^ ^31^, ^32^ ^]^ Cr and Fe do not have any characteristic redox couples in this potential range and therefore excluded.^[^ ^33^ ^]^ The anodization profile to modify the films is shown in Figure 4b. In the first step of native oxide reduction, a negative current is recorded, indicating that a reduction reaction occurs at the sample surface. The −400 V films have the highest cathodic current (≈−200 μA cm^−2^). The current for the floating film decreases after 6 min and reaches a current maximum, similarly to −400 V. The second step is the oxidation of the reduced surface at the potential A2 to form the new active sites for catalysis. The current in this step is positive for all films, highest for the −400 V film with a plateau around 400 μA cm^−2^ for 10 min. To further activate the films, 200 CVs were recorded from 0 to 0.6 V versus Ag/AgCl at a high scan rate (100 mV s^−1^). The final 200^th^ CV is shown in Figure 4c.

**Figure 5:**
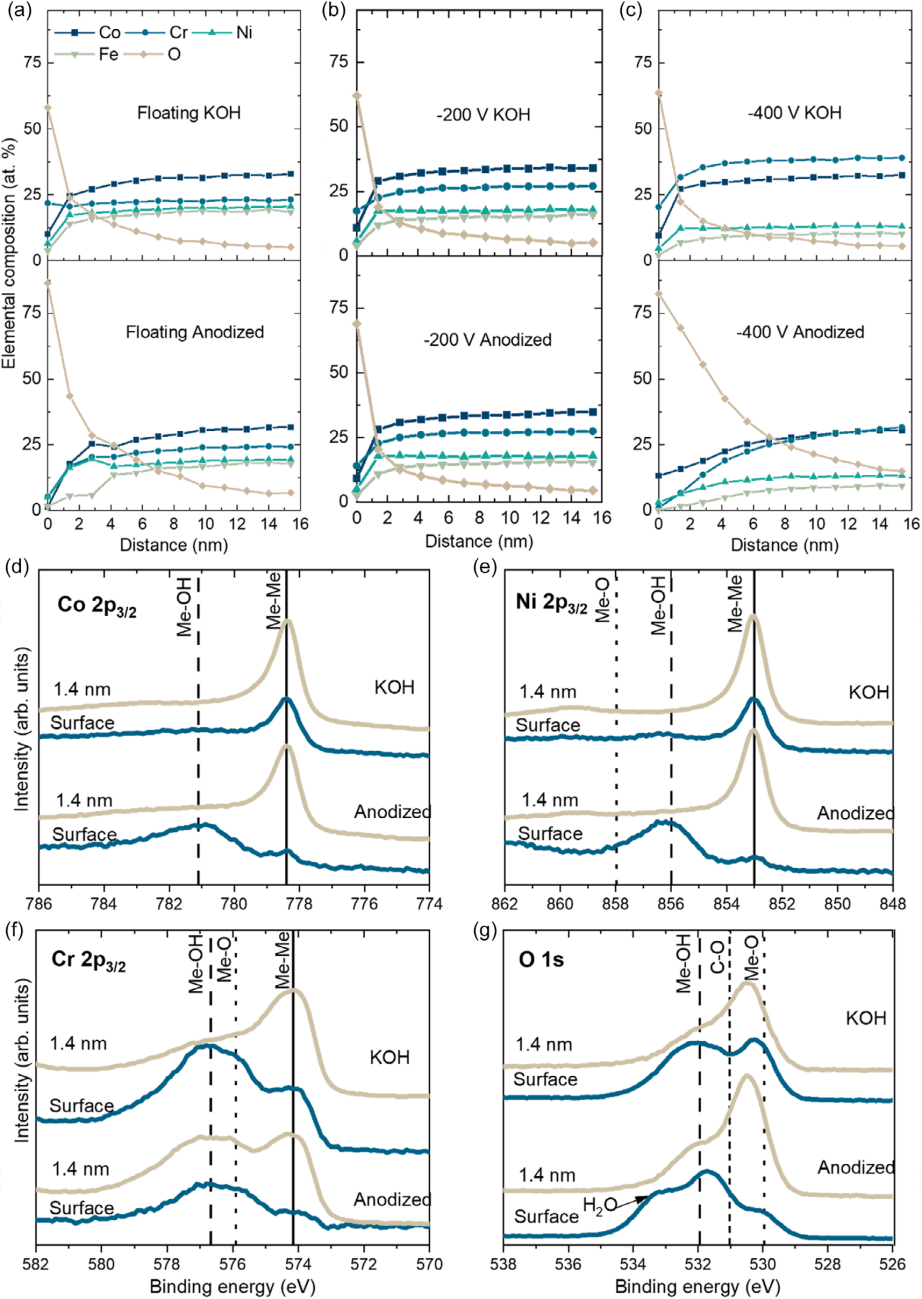
XPS depth profiles of a) floating, b) −200 V, and c) −400 V films exposed to KOH for 30 min and after anodization. Floating film core level spectra d) Co 2*p*_3/2_, e) Ni 2*p*_3/2_, f) Cr 2*p*_3/2_, and g) O 1*s*, metals in full lines, hydroxides in dashed lines and oxides dotted lines.

X‐ray photoelectron spectroscopy (XPS) depth profiles of the anodized films were recorded and are shown in **Figure** **5**. For comparison, films immersed in KOH for 30 min (the as‐deposited conditions for catalysis testing) were also analyzed. The metallic spectra were normalized using the Me–Me peak and the O 1*s* spectra normalized at BE = 532 eV. For as‐deposited films exposed to KOH, the oxygen content decreases rapidly with depth, which is typical for a native oxide layer (2–4 nm). The high Cr content in comparison to the other metals indicates the formation of a Cr‐rich oxide. The anodized floating and −400 V films have a much thicker oxide layer, around 8–10 nm. The difference between the as‐deposited and the anodized state for the −200 V films is not as evident. In the floating and −400 V anodized films, there is a slight Co and Ni enrichment and a clear lower Fe content in the oxide up to 4 nm in depth. Peak fitting of the Co 2*p*_3__/__2_, Ni 2*p*_3__/2_ and O 1*s* revealed that the surface of the anodized films has Co^2+^, Ni^2+^ bonded to OH^−^ (dashed lines in Figure 5) which indicates the presence of Ni and Co hydroxides. In Ni 2*p*_3__/2_ there is also a signal from Ni^3+^, which is associated with NiOOH. This was confirmed by XRD, shown in the Figure S4, Supporting Information, with the presence of an additional peak in the diffractogram corresponding to a mix of Ni(OH)_2_ and Co(OH)_2_.^[^ ^34^, ^35^, ^36^ ^]^ The signal for hydroxide bonds was weaker with depth, indicating that it is a metal oxide underneath the surface hydroxide layer.

**Figure 6:**
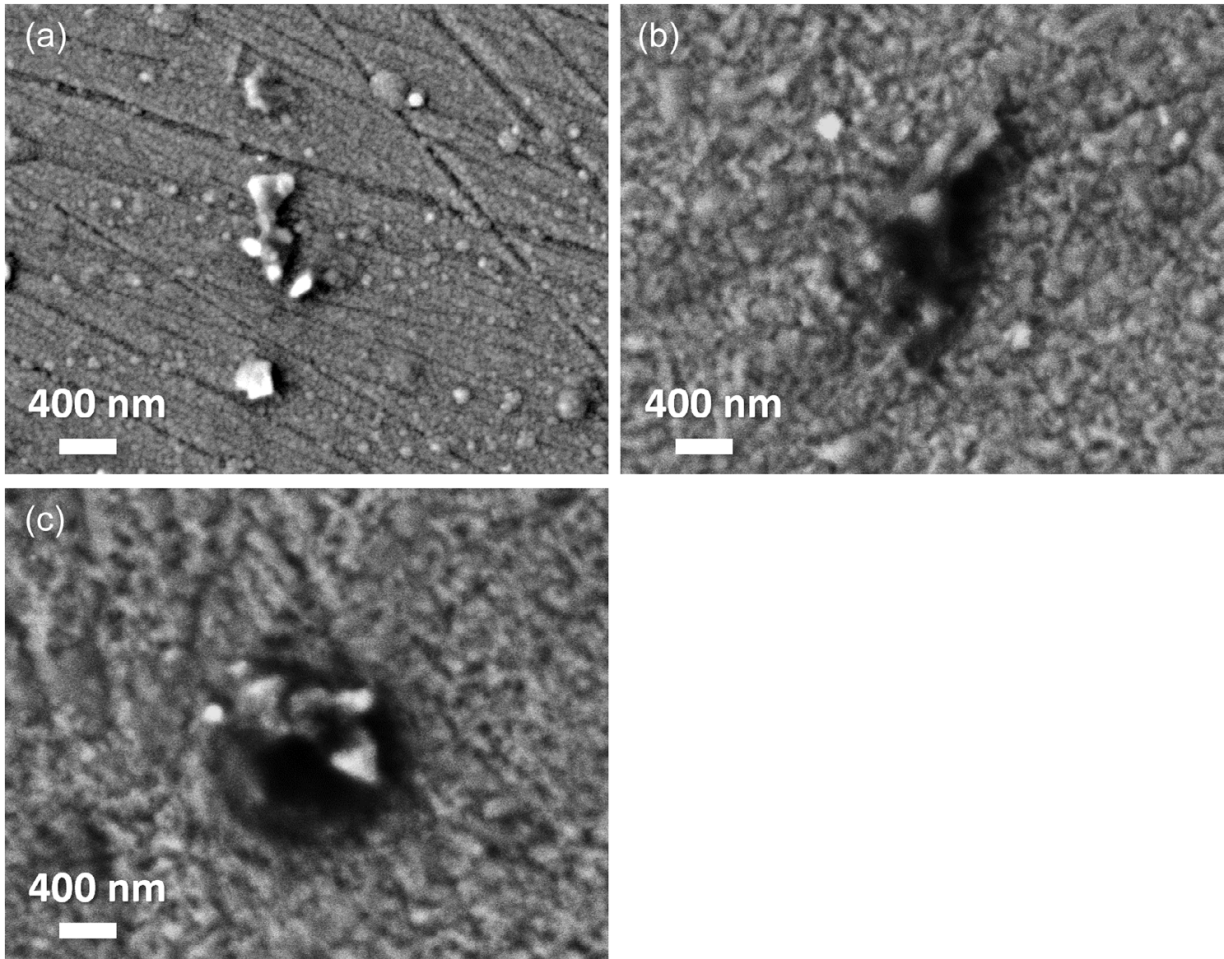
SEM images of anodized films a) floating, b) −200 V, and c) −400 V.

Films after anodization were also analyzed by SEM‐EDS and the overall O content increased for the films. Particles with uneven shapes had formed on the surface with an even higher O content, see **Table** **2** which lists the compositions measured by EDS in the SEM images in **Figure** **6**.

**Table 2:** EDS analysis of particles and surrounding films of areas shown in Figure 6.

	At%	O	Cr	Fe	Co	Ni
Floating	Particles	16.7 ± 2.4	17.5 ± 2.9	20.9 ± 1.2	25.0 ± 1.2	19.8 ± 1.2
	Surrounding film	4.4 ± 0.4	18.9 ± 0.5	20.6 ± 1.6	30.8 ± 0.4	25.9 ± 0.4
−200 V	Particles	22.3 ± 9.8	17 ± 1.1	13.9 ± 2.8	27.4 ± 4.5	19.4 ± 1.8
	Surrounding film	4.7 ± 0.7	22.9 ± 2.9	17.7 ± 1.3	31.7 ± 1.3	22.9 ± 1.2
−400 V	Particles	49.4 ± 10.9	19.1 ± 4.3	5.9 ± 1.4	16.3 ± 3.5	9.3 ± 2.3
	Surrounding film	2.0 ± 0.5	29.2 ± 0.1	13.2 ± 0.4	35.3 ± 0.5	20.3 ± 0.3

### 2.3 Electrocatalytic Activity

**Figure 7:**
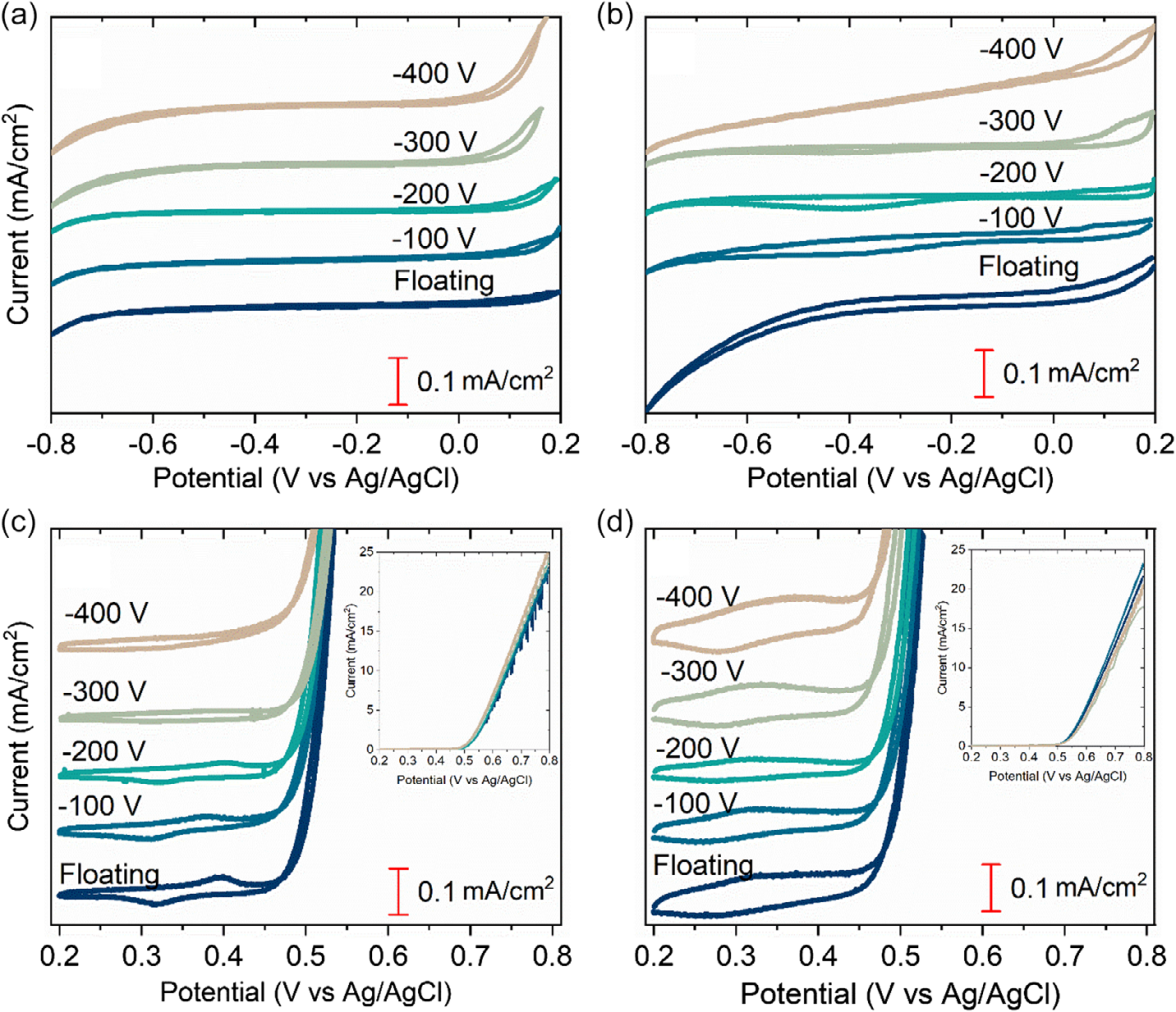
Cyclic voltammograms (plotted with y‐offsets) recorded in ORR a,b) and OER potential regions c,d) with inserts for the current densities up to 10 mA cm^−2^. As‐deposited films are shown in (a,c). Anodized films shown in (b,d).

The catalytic activity of the as‐deposited and anodized films in ORR and OER was evaluated in 1 m KOH saturated with O_2_. CVs were recorded with a scan rate of 5 mV s^−1^ and are shown in **Figure** **7**.

**Figure 8:**
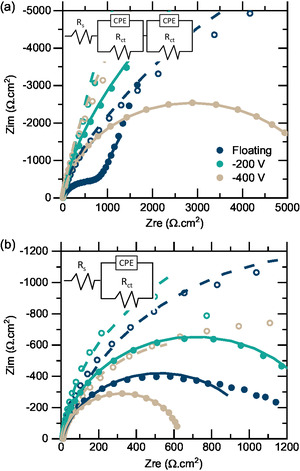
Nyquist plots of EIS data recorded close to a) onset potential of ORR (−0.1 V) for as‐deposited films (non‐filled circles) and anodized films (filled circles); b) onset potential of OER (0.45 V) for as‐deposited films and anodized films.

For ORR, the as‐deposited films have a current increase around −0.8 V, indicating that the catalytic activity is very low and the overpotential for ORR high. The current peak is associated with the onset of ORR as it is not seen in the oxygen free (purged with N_2_) environment (Figure S5, Supporting Information). The anodized films have a current increase around −0.25 V. For OER, both the as‐deposited and anodized films are active as the current density increases sharply around 0.45 V. At 0.8 V the current density reaches 25 mA cm^−2^. For anodized films, the shape of the CV changes after anodization, for example, the peak before the onset of OER (around 0.4 V) disappears. The ORR and OER kinetic information of the films were evaluated by their Tafel slopes and their onset potential, shown in **Table** **3**. Additionally, EIS was recorded close to the onset potential of ORR and of OER (−0.1 and 0.45 V vs Ag/AgCl respectively). The data was fitted and is shown together with the raw data in Nyquist plots, see **Figure** **8**. The extracted charge transfer resistance is shown in Table 3. For ORR, it was attributed to the resistance of the outer layer in the double layered model with two R‐CPE circuits in series. For OER, it was attributed to the resistance of the single R‐CPE circuit.

**Table 3:** Tafel slope, onset potential and charge transfer resistance extracted from fitted EIS data. The equivalent circuit used for the fit is a single or double Randles circuit with a CPE.

		ORR			OER		
		Tafel slope [mV dec^−1^]	Onset potential [V]	Charge transfer resistance [Ω cm^2^]	Tafel slope [mV dec]	Onset potential [V]	Charge transfer resistance [Ω cm^2^]
Floating	As‐deposited	476	−0.67	1 463	52	0.54	2 897
	Anodized	366	−0.25	774	51	0.53	1 032
−100 V	As‐deposited	452	−0.69	1 180	48	0.53	2 075
	Anodized	318	−0.20	5 264	49	0.53	1 291
−200 V	As‐deposited	632	−0.72	2 812	67	0.54	2 903
	Anodized	193	−0.16	2 573	45	0.53	1 403
−300 V	As‐deposited	552	−0.55	4 903	61	0.54	852
	Anodized	203	−0.19	662	45	0.52	717
−400 V	As‐deposited	531	−0.52	8 917	59	0.54	1 403
	Anodized	132	−0.26	880	56	0.51	625

For as‐deposited films, the Tafel slopes for ORR and OER are the lowest for the −100 V film (452 and 48 mV dec^−1^) and highest for the −200 V film (632 and 67 mV dec^−1^). After anodization, the Tafel slopes for ORR are decreasing with increasing substrate bias, whilst for OER they are remain in the same range or slightly decreasing. For as‐deposited films the ORR onset potential is lower for the high bias films (−300 and −400 V) than the low bias films. After anodization, all films have a lower onset potential, with values around −0.2 V versus Ag/AgCl. For OER the onset potentials for as‐deposited films are very similar (0.54 V) and a small decrease was observed after anodization for the −300 and −400 V films (0.51–0.52 V). The ORR charge transfer resistance of as‐deposited films increases with substrate bias whereas for OER charge transfer resistance decreased. After anodization, the charge transfer decreased for all films for both reactions, and the biggest differences are seen for the floating, −300 and −400 V films. Overall, the Tafel slopes, onset potentials, and charge transfer resistances decrease for both ORR and OER after anodization in comparison to the as‐deposited films. The most significant difference between as‐deposited and anodized films is seen for the floating and −400 V films. The ORR potentials increased after anodization from 0.3 to 0.7 V versus RHE, that is, reducing the overpotential from 0.9 to 0.5 V, which is 0.1 V higher than the Pt/C + Ir/C reference noble catalyst in Chen et al.^[^ ^37^ ^]^ study. For OER, all potentials are in the range of 1.5 V versus RHE, that is, 0.3 V overpotential, which is similar to the values reported for Ir/C.^[^ ^38^ ^]^

**Figure 9:**
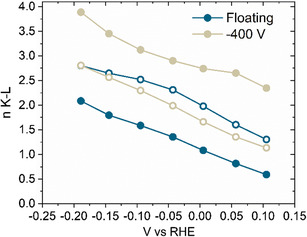
Number of electrons transferred during ORR versus potential for Floating and −400 V film. Full circles represent anodized films and non‐filled circles are the as‐deposited films.

To determine the ORR pathway taken by the floating and −400 V films, a Koutecky–Levich analysis was carried out with a rotating ring disc electrode. The number of transferred electrons could then be extracted, and the results are shown in **Figure** **9**.

For all films the number of electrons increases with potential as the overpotential for ORR decreases. The as‐deposited films have a maximum value for n of 2.8 electrons. After anodization, n decreased to 2.1 electrons for the floating film and increased to 3.9 electrons for the −400 V film. This indicates a change in the ORR pathway after anodization.

### 2.4 Durability and Corrosion Resistance

**Figure 10:**
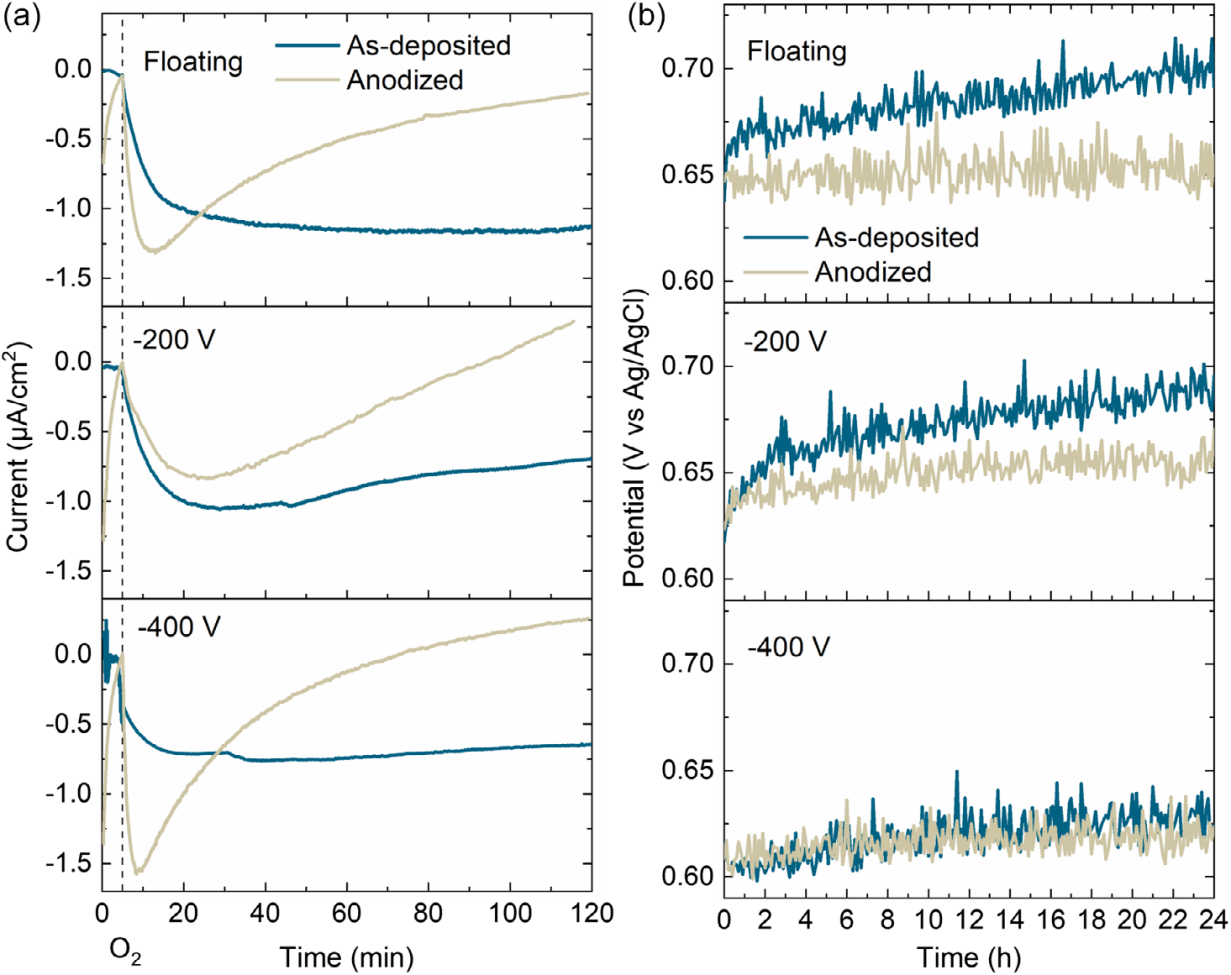
ORR and OER testing over time. For ORR a), the current density was measured at applied potential of −0.4 V vs Ag/AgCl over 2 h, O_2_ gas was introduced after 5 min of the measurement. b) Durability of OER catalysts investigated by measuring the potential at applied current density of 10 mA cm^−2^ over 24 h.

ORR and OER performance over time before and after anodization was evaluated for the floating, −200 and −400 V films, and is shown in **Figure** **10**. The ORR performance of the films was evaluated by applying −0.4 V (vs Ag/AgCl), based on Figure 7, and by recording the current density until it had stabilized or decreased significantly. The ORR current density was extracted by subtracting the current density recorded in the N_2_ purge. For all films the current density increases rapidly when oxygen is introduced, indicating a fast response of the films toward ORR. However, the current densities reached are in the μA cm^−2^ range instead of the mA cm^−2^ range reported for noble catalysts. Nonetheless, for the as‐deposited floating and −400 V film the current densities stabilized around −1.2 and −0.7 μA cm^−2^, respectively. For the other films the current decreases over time, especially for the anodized films. Even though the response of the anodized films is faster than the as‐deposited films, that is, sharper increase in current, the performance over time is less stable.

For OER, the evolution of the potential was recorded when 10 mA cm^−2^ was applied to the films. The lowest potential was measured for the −400 V film to be 0.59 V versus Ag/AgCl (1.45 vs RHE). The highest potential was for the floating film in its as‐deposited state, that is, 0.71 V versus Ag/AgCl. The potential for the as‐deposited floating and −200 V films also increased over time by 77 and 85 mV, respectively. After anodization the increase in potential is no longer observed in addition to lowering the average values of the potential.

The floating and −400 V films were analyzed by SEM‐EDS after 24 h of OER testing. The SEM images and EDS analysis are available in (Figure S6 and Table S1, Supporting Information). There was no significant change in film morphology compared to as‐deposited state (Figure 1) or after anodization (Figure 6). But the −400 V film had a significantly higher O content (18 at%) for the surrounding film, not just the particles in both as‐deposited and anodized state. This indicates that the −400 V films get oxidized during the testing.

**Figure 11:**
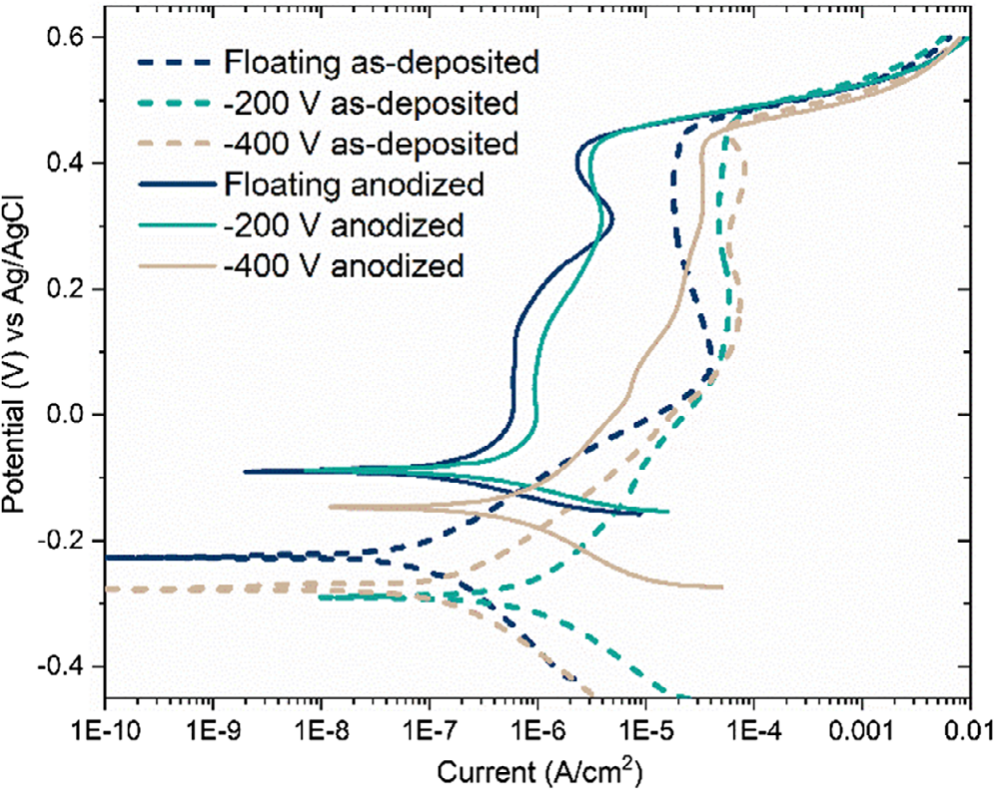
Potentiodynamic polarization curves recorded in 1 m KOH, pH 13 for the floating, −200 and −400 V films as‐deposited (dotted lines) and anodized (full lines).

To ensure the films have a good corrosion resistance in a fuel cell or electrolyzer environment, potentiodynamic polarization curves were recorded. The results are shown in **Figure** **11**.

**Figure 12:**
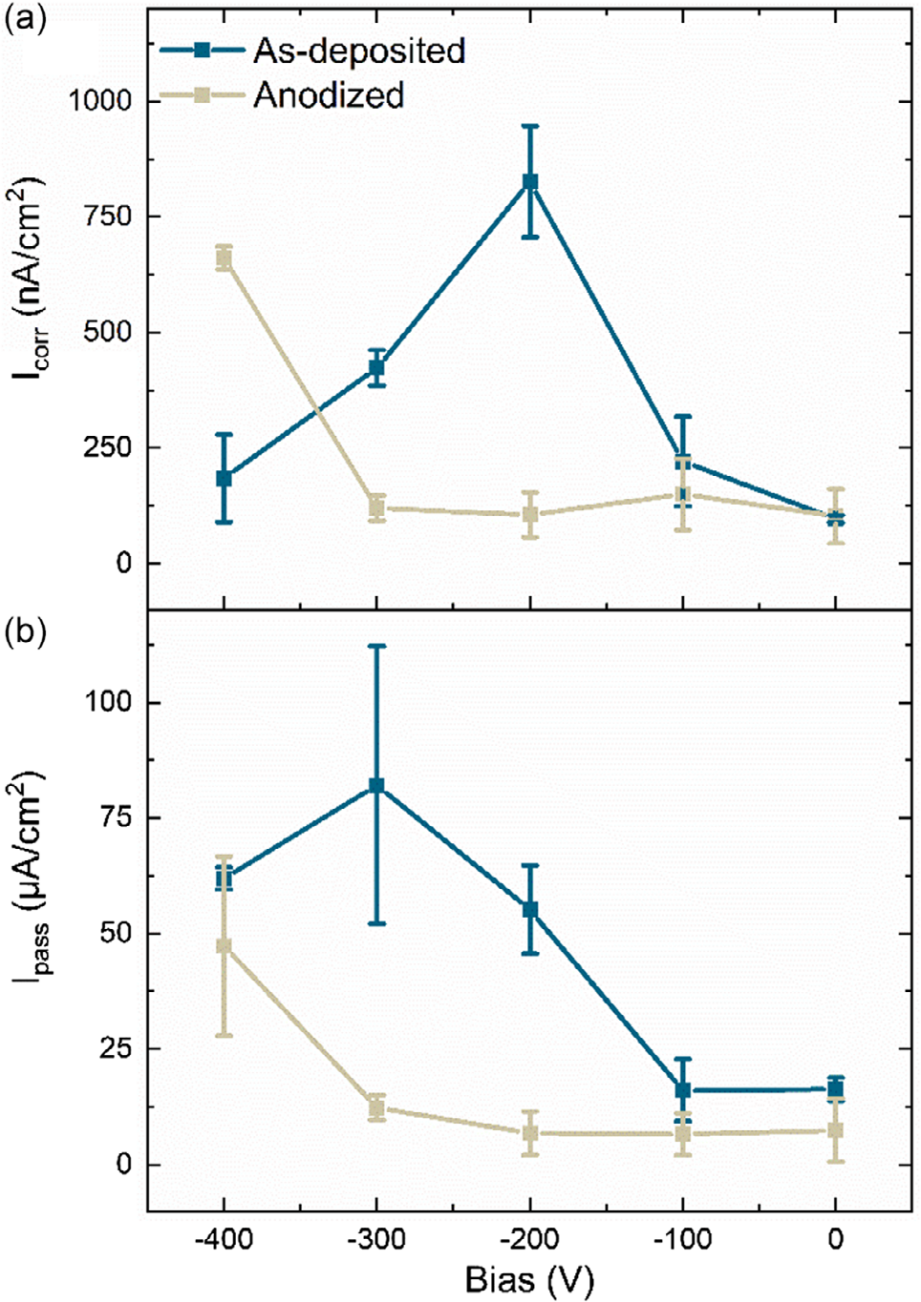
a) *I*_corr_ and b) *I*_pass_ for the as‐deposited and anodized films versus applied substrate bias.

Above the corrosion potential (around −0.3 V for as‐deposited, −0.1 V for anodized films) the current reaches a plateau corresponding to the passive region. Above 0.5 V, the current increases rapidly as a result of the breakdown of the passive film and the OER which also contributes to the current density. An additional peak around 0.3 V is present for some of the films, which is the same peak identified in the anodization and activation step (Figure 4a). The corrosion current density (*i*_corr_) was extracted with Tafel extrapolation, shown in **Figure** **12**a. The corrosion current density is more than doubled when a −100 V substrate bias is applied and even higher for the −200 V bias. *i*_corr_ reaches a maximum value for the −200 V film as it decreased again for the −300 and −400 V films. The floating HiPIMS films have a 25 times lower corrosion current density than the equivalent floating DC magnetron sputtering films in our previous study.^[^ ^30^ ^]^ The passive current densities are different for the films and are shown in Figure 12b. An increase of the passive current density *I*_pass_ with increasing bias is also observed. The maximum value is observed for the −300 V film.

For anodized films, the reversed V‐shaped trends for the corrosion and passive current densities against substrate bias are no longer visible. Both current densities for anodized films are lower than the as‐deposited films for all films except for the −400 V film.

## 3 Discussion

### 3.1 Effect of Substrate Bias on Film Structure

Applying substrate bias during the HiPIMS depositions affected the film composition, crystal structure, grain size, and growth morphology. The change in composition, that is, increase in Co and Cr content and decrease in Fe and Ni, is attributed to re‐sputtering of deposited atoms due to the high‐energy incident species.^[^ ^39^ ^]^ The different compositions, in turn affected the crystal structure, for example, the higher Co content in −400 V film led to the formation of additional HCP phase. The formation of the secondary sigma phase in the floating and −100 V films is attributed to the presence of defects such as stacking faults, as seen in our previous study.^[^ ^40^ ^]^ The ion bombardment also increased the residual stress in the films as a shift of the main FCC peak in the X‐ray diffractograms was observed.^[^ ^16^, ^41^ ^]^ Resputtering also affected the film thickness as it decreased with increasing bias when the resputtering effect is more pronounced due to increasing sputter yields with increasing ion acceleration.^[^ ^42^ ^]^

The beneficial effect of the ion bombardment during deposition is the densification of the films.^[^ ^16^, ^42^, ^43^, ^44^ ^]^ Indeed, in the electron microscopy analysis shown in Figure 2, the typical columnar structure of thin films observed for the film deposited at floating potential is not seen for the films deposited with bias and the domain sized in the band contrast images increases as well. The grain size increases from 60 nm for the film deposited at floating potential to 150 nm for film with −100 V bias. Further increase of the bias value results in irregular and faceted surface rather than an increase in grain size. Both variations in the films structure are attributed to higher energy of bombarding ions with high substrate bias. This affects adatom mobility which will allow formation of larger grains and cellular structures rather than columns until a renucleation point is reached.^[^ ^16^ ^]^ This is the opposite of what has been seen in literature for AlCrTiVZr and TiN films.^[^ ^16^, ^42^ ^]^ These studies were however carried out with no external heating during the depositions. However, for Cu films without intentional heating similar grain growth trends as in this study were observed.^[^ ^17^ ^]^ For TiN/Cr_2_N deposited at 350 °C, a small increase in grain size was observed for bias values higher than −100 V.^[^ ^41^ ^]^ For CoCrFeNiN films deposited at 300 °C, increasing the bias resulted in grain growth.^[^ ^45^ ^]^ Thus, the discrepancy with previous reports is attributed to the combination of high ion bombardment, heating of the substrates, which also provides higher adatom mobility,^[^ ^46^ ^]^ and the material system itself.

### 3.2 Bifunctional Catalysis toward Oxygen Reactions

The as‐deposited and anodized films did show some activity for both ORR and OER, but the performance toward OER was considerably higher than for ORR, in terms of overpotential, current densities and durability. For the as‐deposited films, the active sites for catalysis, that is, Co and Ni ions were detected by XPS, but in far lower relative intensity compared to the anodized films. This would explain the lower catalytic activity of the as‐deposited films. After reducing the native oxide, the surfaces of the films were further oxidized by anodization to the desired catalytically active species, which did improve the electrocatalytic performance of the films. This improvement is attributed to the enrichment in Co and Ni in the oxide after anodization which provides more of the active sites Co^2+^, Ni^2+^, and Ni^3+^, which have been reported for both ORR and OER.^[^ ^28^, ^29^, ^32^ ^]^ As more active sites are available, there is better electron transfer in the anodized films and the overpotential is reduced. For the ORR Tafel extrapolation, only one slope was considered at high overpotential values, resulting in a one‐step Tafel slope for our catalysts. Overall, our Tafel slopes differ from the theoretical values.^[^ ^47^ ^]^ Higher Tafel slope values have been observed for Pt in alkaline electrolytes compared to acidic electrolytes.^[^ ^47^, ^48^ ^]^ In the theoretical values the coverage of the active sites by intermediate species is either 0 or 1,^[^ ^47^ ^]^ which might not be the case in practice and thus introduce a source of error. Nonetheless, for anodized films deposited with high bias values, the slopes are approaching the 120 mV dec^−1^ theoretical values where the first electron to the adsorbed oxygen molecule is the rate determining step.^[^ ^47^ ^]^

Rotating disk electrode measurements were carried out to determine the ORR pathway based on the number of transferred electrons *n*. The as‐deposited films have a maximum n value of 2.8, which would suggest either a 2‐electron transfer with hydrogen peroxide as an intermediate product, or a (2 + 1) electron transfer. The latter would imply that after its formation, the hydrogen peroxide reacts with an active site in a Fenton‐like reaction to produce a hydroxide radical. The active site is then regenerated by an additional transfer.^[^ ^49^, ^50^ ^]^ The (2 + 1) pathway equations mentioned earlier are given below for Fe^2+^/Fe^3+^ as Fe is the more common catalyst for the Fenton reaction. A 3 e^−^ ORR pathway has however also been reported for FeCo, CoSe_2_/Cu, NiFeO_*x*_, and NiO and often referred to the Fenton as a reaction intermediate.^[^ ^50^, ^51^, ^52^, ^53^ ^]^ It is therefore likely that the CoCrFeNi films as‐deposited follow that pathway as well. For the anodized floating film n is 2.1 and for the anodized −400 V film n is 3.9, thus a 2‐electron ORR and 4‐electron ORR pathway respectively. A summary of the reaction pathways is given below: 1) $O_{2}{\text{+2H}}_{2}{\text{O + 2e}}^{-}\to H_{2}O_{2}{\text{+ 2OH}}^{-}\left({\text{2 e}}^{-}\text{ ORR}\right)$; 2) $\begin{array}{c} H_{2}O_{2}{\text{ + Fe}}^{\text{2+}}\to {\text{OH• + Fe}}^{\text{3+}}{\text{+ OH}}^{-}\left(\text{Fenton}\right){\text{ Fe}}^{\text{3+}}{\text{+ e}}^{-}\to {\text{Fe}}^{\text{2+}}\left({\text{+1 e}}^{-}\text{, regeneration of active sites}\right) \end{array}$; 3) $O_{2}{\text{ + 2H}}_{2}{O\text{ + }\text{4e}}^{-}\to {\text{4 OH}}^{-}\left({\text{4 e}}^{-}\;\text{ORR}\right)$

After anodization of the floating film, the regeneration of the active sites after the Fenton reaction is likely no longer happening, which could be related to the lower Fe content at the film's surface or irreversible changes in the active sites structure. For the −400 V film the presence of more active sites allows a higher number of electrons to be transferred. The change in performance after anodization was also seen for the ORR durability tests. The current density at −0.4 V reached a higher maximum, but decreased considerably after anodization, which could indicate that the active sites were consumed and not stable enough. Performance loss could also be due to change in the structure over time, which has been observed for Pt/C catalysts where corrosion of the carbon support was assigned as the main cause.^[^ ^54^ ^]^

### 3.3 Corrosion Resistance

All films have a high Cr content (>20 at%) and Cr detected in the oxide film as Cr_2_O_3_ and Cr(OH)_3_ which indicates the formation of a Cr containing passive film that protects the films from corrosion and their passive behavior.^[^ ^55^ ^]^ There are differences in corrosion resistance and are explained by the difference in grain size and film morphology. It has been shown that grain refinement can be beneficial for corrosion resistance of stainless steels in NaCl environments^[^ ^19^ ^]^ and for Co coatings.^[^ ^20^ ^]^ For refined microstructure the grain boundary density is higher than a coarse grained material, which can improve the repassivation kinetics of the material.^[^ ^20^ ^]^ As the grain size increases for the −100 and −200 V film compared to the floating film then corrosion resistance decreases. The corrosion resistance increases again when the bias is further increased to −300 and −400 V. Here the densification and the complete suppression of the columnar growth with defects such as stacking faults could be the reason for the better corrosion performance.^[^ ^56^, ^57^ ^]^

The anodized films have a lower corrosion current density than the as‐deposited films, only the −400 V film shows a significant increase in corrosion rate. The improvement is attributed to the formation of a thicker oxide, which is known to protect alloys against corrosion, that is, aluminum alloys.^[^ ^58^ ^]^ In the case of the −400 V film the presence of the HCP phase in the bulk of the film could cause a galvanic coupling and with the anodized oxide if it is porous. The detrimental effect of dual phases in HEAs on the corrosion resistance in NaCl environments has been seen before.^[^ ^26^, ^30^ ^]^

## 4 Conclusions

CoCrFeNi thin films were synthesized as thin films on steel substrates by HiPIMS. The grain size, density, and structure of the films were significantly affected by a DC substrate bias, that is, the grain size increased, multiple phases formed, and defects were present as a result of the higher energies of the bombarding ions with increasing substrate bias.

Anodization of the films resulted in enrichment in active sites, that is, Co^2+^, Co^3+^, Ni^2+^, and Ni^3+^ on the films’ surface which increased the ORR and OER activity. All films showed catalytic activity for both reactions; however, the anodized films outperformed the as‐deposited films, in particular for ORR. It was also observed that the ORR pathway changed after anodization. Nonetheless, the OER activity of the films was considerably higher than ORR. The films were also found highly stable for OER over 24 h of testing.

Finally, the films were also found to have a high corrosion resistance in alkaline media. The high corrosion resistance of the films was attributed to the small grain size and high density of the films. These non‐precious 3*d* transition metal films show great potential for corrosion resistant oxygen electrocatalysts.

## 5 Experimental Section

5.1

5.1.1

##### Thin Film Synthesis

CoCrFeNi films were deposited on polished carbon steel substrates using HiPIMS. An ultra‐high vacuum system was used with a base pressure in the chamber <10^−6^ Pa. Three targets were used in confocal configuration: a CrNi compound target (3 mm thick), an elemental Co target (3 mm thick), and an Fe elemental target (1 mm thick) all from Kurt J Lesker, USA. Argon was used as working gas with a pressure of 0.4 Pa (3 mTorr). The targets were powered by three separate HiPIMS units (Ionautics, Sweden) fed by three separate DC units (see schematic in Figure S1, Supporting Information). All HiPIMS units were controlled by a synchronizing unit, enabling the simultaneous start of the pulses. The negative discharge pulse length was 40 μs at 500 Hz frequency. The target discharge voltage and current characteristics measured on the respective targets are shown in Figure S1, Supporting Information.

Low‐alloyed carbon steel (C10E) mirror polished plates were used as substrates. Before the depositions, the substrates were ultrasonically cleaned in acetone and isopropanol. The substrate was heated to 300 °C prior to and during the deposition. Five different DC substrate bias cases were studied: floating (no bias applied), −100, −200, −300, and −400 V.

##### Structural Characterization

The crystal structure of the films was characterized by XRD using a Bruker D8 Discover diffractometer in a symmetric *θ*/2*θ* configuration and Cu Kα X‐ray source (*λ* = 1.5406 Å). The morphology and chemical composition of the thin films were analyzed using SEM Sigma 300 VP Gemini (Zeiss, 5 kV acceleration voltage), and an EDS detector (Oxford Instruments X‐MAx^N^, 5 kV acceleration voltage) integrated in the microscope. The average grain size for the films was determined from 20 grain measurements in the SEM images.

Cross‐sectional samples suitable for analysis both by SEM and STEM) were prepared using a dual beam SEM‐focused ion beam (FIB) microscope (Gemini Zeiss 1540 EsB). Pt layers were first deposited on top of the surface of the films to protect them, samples were then tilted to 36° for milling. SEM images of the cross sections were taken using the same instrument using image correction to take account of the sample tilt. Thin lamella (<100 nm) suitable for TEM analysis was prepared using the well‐established lift‐out approach. TEM analysis was performed using a FEI Tecnai G2 TF 20 UT microscope operated at 200 kV acceleration voltage. Dark field STEM images were taken with an angular detector. The crystal texture of selected was further investigated by TKD. This was performed in a Gemini 540 SEM instrument fitted with an Oxford Instruments Symmetry electron backscatter diffraction detector. Samples were tilted to an angle of 20° prior to measurement.

XPS was used for elemental analysis and assessment of chemical bonding. O 1*s*, Co 2*p*, Cr 2*p*, Fe 2*p*, and Ni 2*p* core level spectra were recorded using an Axis Ultra DLD instrument from Kratos Analytical (UK) employing monochromatic Al Kα radiation (*hν* = 1486.6 eV) and an anode power of 150 W. The base pressure during spectra acquisition was lower than 1.5 × 10^−7^ Pa (1.1 × 10^−9^ Torr). No charge compensation was used during analysis. The analyzer pass energy was set to 20 eV resulting in the full width at half maximum of 0.55 eV for the Ag 3*d*_5/2_ peak of sputter‐etched Ag sample used for calibration. All spectra were collected at normal emission angles. The area analyzed by XPS was 0.3 × 0.7 mm^2^. As the commonly used charge referencing method relying on the C 1*s* peak of adventitious carbon is not reliable,^[^ ^59^ ^]^ all spectra were referenced to the sample Fermi edge. For sputter depth profiles, a 0.5 keV Ar^+^ ion beam was used at a 20° incident angle from the surface plane. Spectra deconvolution and quantification was performed using CasaXPS software package (version 2.3.16), sensitivity factors supplied by the instrument manufacturer and Tougaard backgrounds were used.

##### Electrochemical Measurements

The electrocatalytic performance of the films was evaluated for both ORR and OER in 1 m KOH. Electrochemical measurements were carried out with a PARSTAT 3000 A‐DX potentiostat. A three‐electrode set‐up was used where the working electrode was the thin film, an Ag/AgCl saturated with KCl (*E* = 0.197 V vs SHE) was used as reference electrode and a Pt wire as the counter electrode. The films were electrochemically activated by an anodization procedure. The first step of the anodization was applying −1 V versus Ag/AgCl for 30 min to reduce the native oxide. Then an anodic potential (0.38–0.4 V vs Ag/AgCl) for 30 min to reform the oxides on the surface of the films. The anodization potential was determined from cyclic voltammograms recorded after the −1 V step, from 0 to 0.6 V with a scan rate of 5 mV s^−1^. The final step of the anodization was 200 CVs to activate the surface by cycling the films between the different oxidation and reduction reactions at a high scan rate (100 mV s^−1^). ORR and OER testing were carried out in O_2_ saturated 1 m KOH with CVs measured from 0.2 to −0.8 V for ORR and 0.2 to 0.8 V versus Ag/AgCl for OER, both scans at the same scan rate of 5 mV s^−1^. Electrochemical impedance spectroscopy (EIS) was measured for all films at the different stages of anodization and catalysis testing. A perturbation of 10 mV was applied with frequencies from 100 kHz to 10 mHz. The data was analyzed and fitted to equivalent electronic circuits using the software ZSimpWin 3.60. Due to high noise in the low frequency range, only the 100 kHz–100 mHz was considered for the fitting.

To elucidate the ORR mechanism, the films were coated on polished stainless steel inserts for the Rotating disc electrode set‐up provided by Pine instruments. A Bio Logic Science SP‐300 potentiostat was used for the measurements. A three‐electrode set‐up was used where the working disc electrode was the thin film, an Hg/HgO electrode (*E* = 0.098 V vs SHE) was used as reference electrode and a Pt wire as the counter electrode. The number of electrons n were extracted from the Koutecky–Levich equation.

To investigate corrosion properties of the films, they were immersed in naturally aerated 1 m KOH. The open circuit potential (OCP) was first measured for 30 min. Potentiodynamic polarization (PD) was carried out from −0.25 V vs OCP to 0.6 V vs Ag/AgCl with a scan rate of 1 mV s^−1^. The Tafel module in the software VersaStudio was used to calculate the Tafel slopes and determine the corrosion current density *i*_corr_. The extrapolation was carried out 0.1 V away from *E*_corr_. All measurements were repeated for duplicate samples and carried out at room temperature.

## Conflict of Interest

The authors declare no conflict of interest.

## Author Contributions

**Clara Linder**: Conceptualization (equal); Formal analysis (equal); Investigation (equal); Visualization (equal); Writing—original draft (lead); Writing—review & editing (equal). **Robert Boyd**: Formal analysis (equal); Investigation (equal); Visualization (equal); Writing—review & editing (equal). **Grzegorz Greczynski**: Formal analysis (equal); Investigation (equal); Writing—review & editing (equal). **Mikhail Vagin**: Formal analysis (equal); Investigation (equal); Writing—review & editing (equal). **Daniel Lundin**: Conceptualization (equal); Formal analysis (equal); Investigation (equal); Visualization (equal); Writing—review & editing (equal). **Karin Törne**: Conceptualization (equal); Supervision (equal); Visualization (equal); Writing—review & editing (equal). **Per Eklund**: Conceptualization (equal); Funding acquisition (equal); Supervision (equal); Visualization (equal); Writing—review & editing (equal). **Emma M. Björk**: Conceptualization (equal); Funding acquisition (equal); Supervision (lead); Visualization (equal); Writing—review & editing (equal).

## Supporting information

Supplementary Material

## Data Availability

The data that support the findings of this study are available from the corresponding author upon reasonable request.
