# Supplementary material for: Enhanced Oxygen‐Reaction Electrocatalysis and Corrosion Resistance of CoCrFeNi Thin Films by Tuned Microstructure and Surface Oxidation
Source: Small Sci. 2024 Sep 29;4(11):2400296. doi: 10.1002/smsc.202400296 (PMC11935288; doi:10.1002/smsc.202400296)
Supplement: Supplementary file 1 — Supplementary Material [file SMSC-4-2400296-s001.pdf]

Supporting Information for **Enhanced oxygen-reaction electrocatalysis and corrosion resistance of CoCrFeNi thin films by tuned microstructure and surface oxidation**

Clara Linder[1,2,*], Robert Boyd[2], Grzegorz Greczynski[3], Mikhail Vagin[4], Daniel Lundin[5], Karin Beaussant Törne[1], Per Eklund[3,6], Emma M. Björk[2]

*[1] RISE, Corrosion, Vehicles and Surface Protection, 164 40 Kista Sweden*

*[2]Nanostructured Materials, Department of Physics, Chemistry and Biology (IFM), Linköping University, 581 83 Linköping, Sweden*

*[3]Thin Film Physics Division, Department of Physics, Chemistry, and Biology (IFM), Linköping University, 581 83 Linköping, Sweden*

*[4]Laboratory of Organic Electronics, Department of Science and Technology, Linköping University, 601 74 Norrköping, Sweden*

*[5]Plasma and Coatings Physics Division, Department of Physics, Chemistry, and Biology (IFM), Linköping University, 581 83 Linköping, Sweden*

*[6]Department of Chemistry – Ångström, Uppsala University, 751 21 Uppsala, Sweden*

**Corresponding author. e-mail:* clara.linder@ri.se

Figure S1 shows a schematic of the PVD deposition system and  the HiPIMS discharge for the films for one pulse length for each target. The black curves are the voltage and the red curves the current. A small fluctuation in the voltage for NiCr is recorded when the current starts to increase.

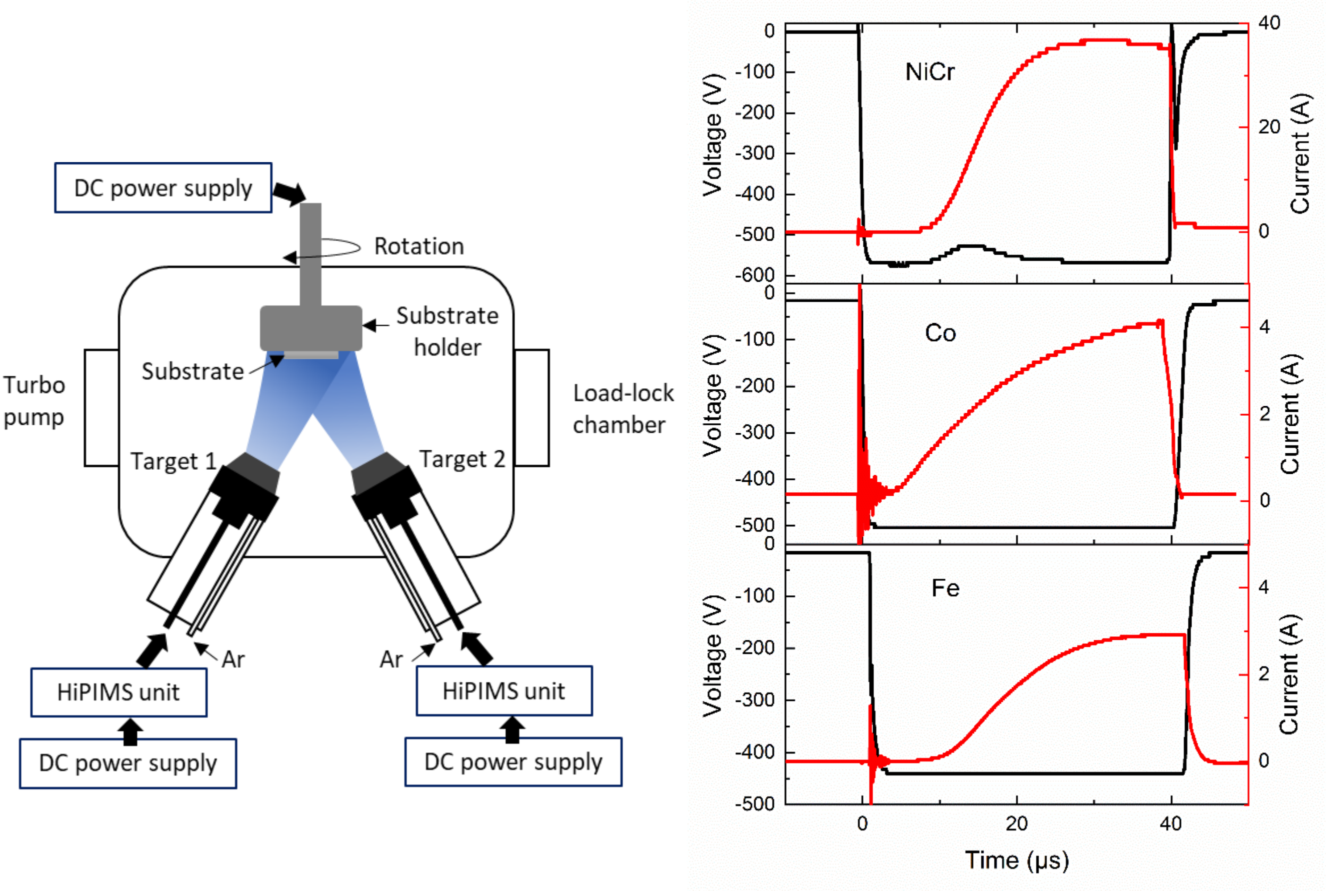


*Figure S1: Schematic of PVD deposition system and HiPIMS discharge voltage-current characteristics for all targets during one 40 μs pulse. Black curve =voltage. Red curve = current.*

Figure S2 shows HAADF STEM images of the film deposited at floating potential. Horizontal features in the columnar structure correspond to stacking fault defects. The voids between the columns corresponds to underdense grain boundaries.

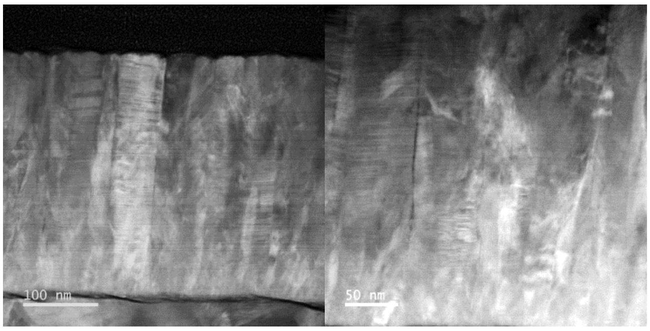


*Figure S2: HAADF STEM image of stacking faults and underdense grain boundary in floating film.*

Figure S3 shows the EDS maps recorded by STEM for the films deposited at floating potential, -200 V, and -400 V bias.

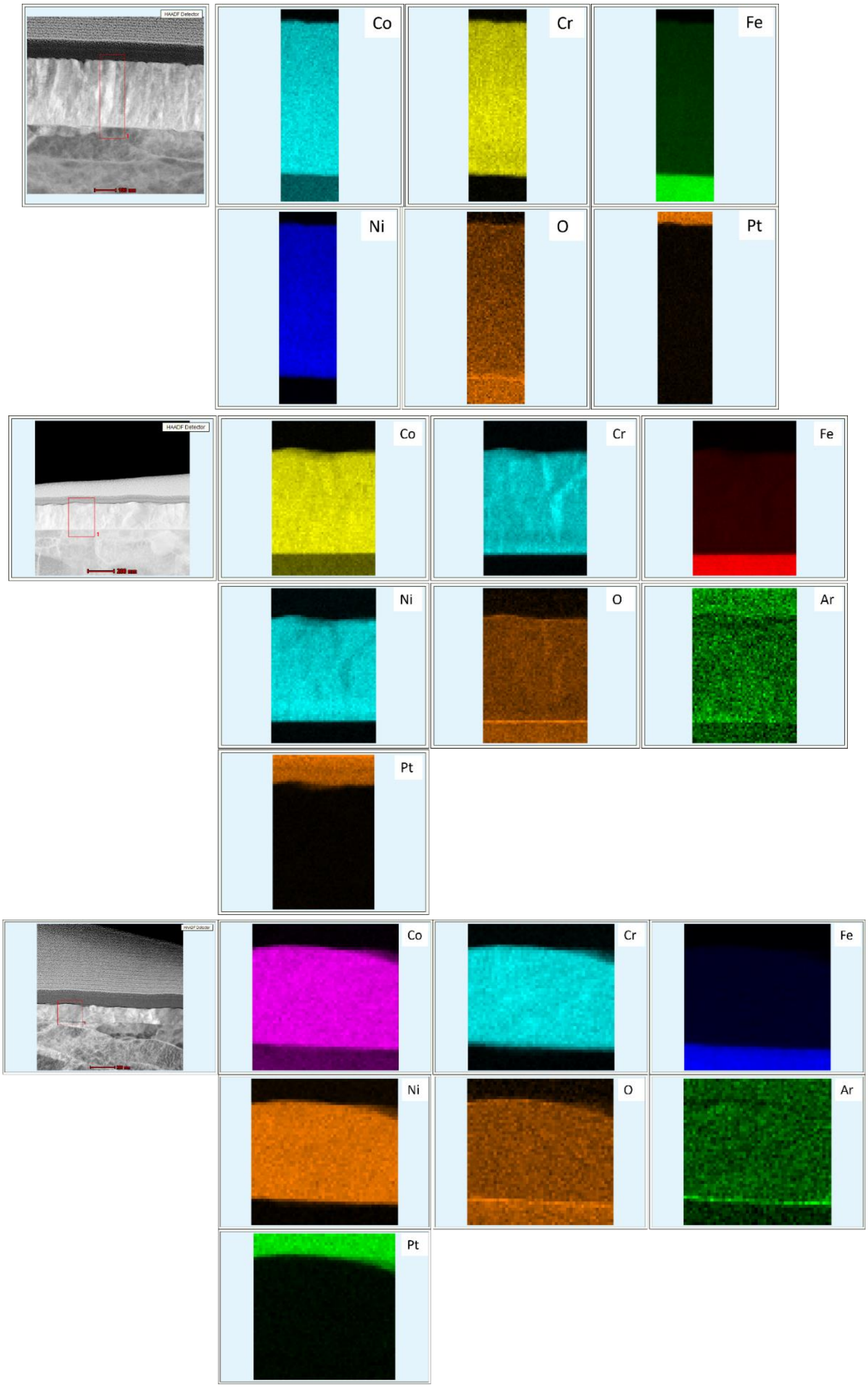


*Figure S3: STEM EDS mapping of films deposited at floating potential, -200 V and -400 V bias.*

Figure S4 shows X-ray diffractograms of films after anodization and activation step.

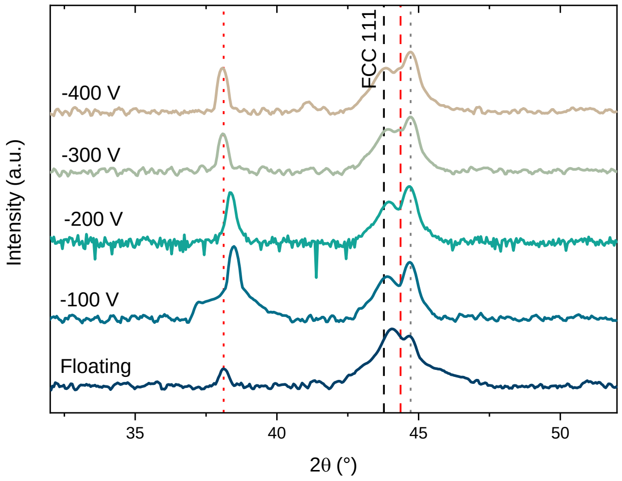


*Figure S4: X-ray diffractograms of films after anodization and activation step.*

Additional peaks are present in the diffractograms from the anodized films compared to the as-deposited films (Fig. 3 in the main paper). The (002) reflection of β-hydroxide at 38° indicates the presences of  a mix of $Ni(OH)_2$ and $Co(OH)_2$ [1–3]. The films deposited at -300 V and -400 V exhibit an additional peak at 44° which corresponds to the (400) reflection of $Co_3O_4$[4]. The (222) reflexion of $Co_3O_4$ is at 38.5°, thus overlapping with the hydroxide peak and not easily distinguished.

Figure S5 shows the CVs for ORR testing recorded in both $O_2$ saturated and $O_2$-free ($N_2$ purged) electrolytes.

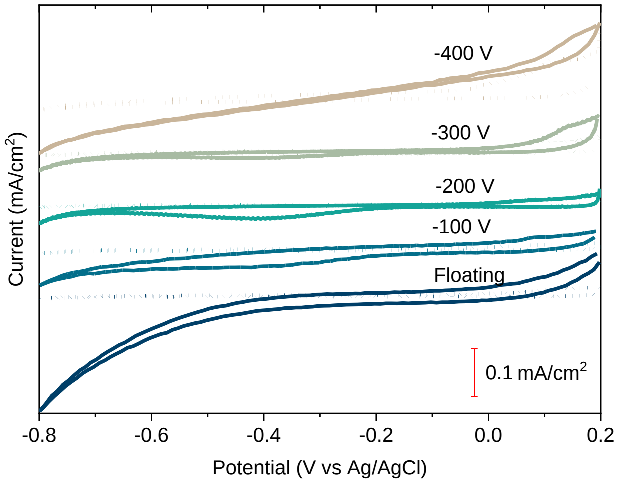


*Figure S5: CVs for ORR testing in 1 M KOH - full lines purged with $O_2$, dotted lines purged with $N_2$.*

Figure S6 shows the floating and -400 V films in as-deposited and anodized state after 24 h of OER testing at 10 mA/cm$^2$.

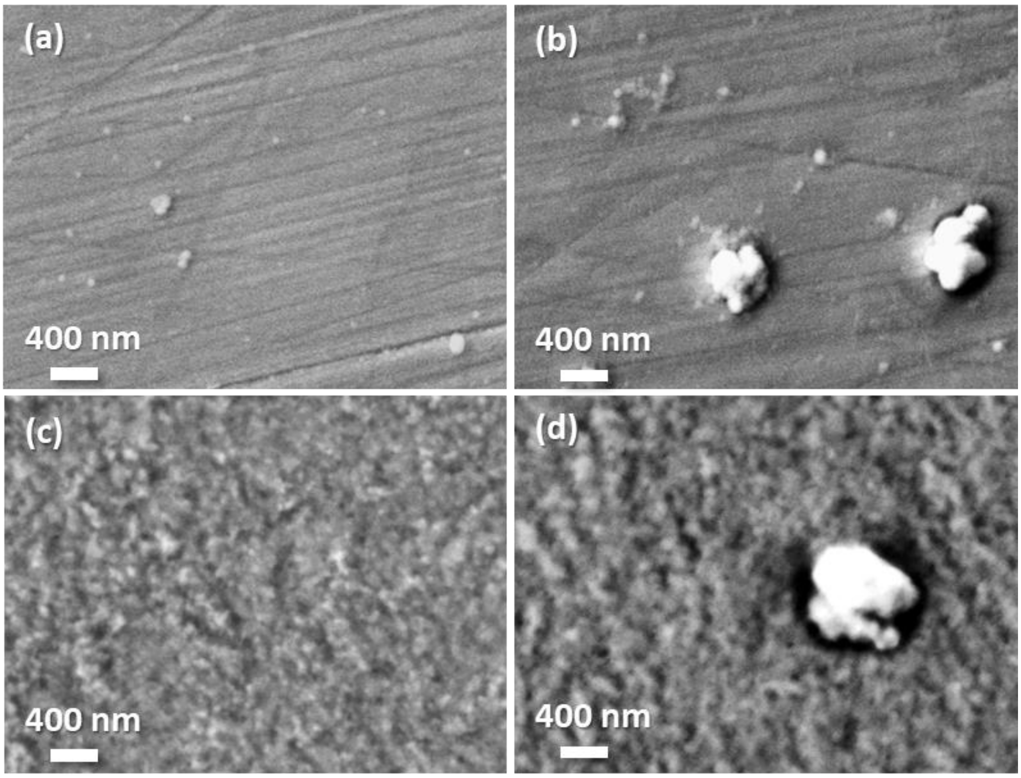


*Figure S6: SEM images of floating as-deposited film (a), floating anodized film (b), 400 V as-deposited film (c), 400 V anodized film (d) after 24 h of OER testing at 10 mA/cm$^2$.*

*Table S1: EDS analysis of films in Figure S6 (at%).*

| | At% | O | Cr | Fe | Co | Ni |
|---|---|---|---|---|---|---|
| **Floating as-deposited** | Film | 3.3 ±0.5 | 21.6 ±1.0 | 19.4 ±0.5 | 30.9 ±0.5 | 24.9 ±0.6 |
| **Floating anodized** | Film | 4.2 ±1.0 | 20.4 ±1.1 | 19.5 ±0.8 | 31.2 ±0.5 | 24.7 ±0.7 |
| **Floating anodized** | Particles | 5.7 ±1.6 | 20.5 ±0.9 | 19.1 ±0.6 | 30.3 ±0.7 | 24.3 ±1.1 |
| **-400 V as-deposited** | Film | 18.5 ±0.0 | 24.7 ±0.3 | 12.6 ±0.4 | 28.8 ±0.2 | 15.5 ±0.5 |
| **-400 V anodized** | Film | 16.1 ±0.4 | 24.1 ±0.9 | 12.3 ±0.6 | 30.1 ±0.3 | 17.4 ±0.5 |
| **-400 V anodized** | Particles | 18.7 ±4.8 | 22.4 ±2.2 | 13.0 ±1.3 | 29.0 ±1.3 | 16.9 ±1.6 |

Table S2 shows the corrosion parameters extracted with a Tafel extrapolation from the polarization curves in Figure 11.

*Table S2: Corrosion potential $E_{corr}$, corrosion current density $i_{corr}$ extracted from potentiodynamic polarisation in Figure 11.*

| | **1 M KOH** | **Floating** | **-100 V** | **-200 V** | **-300 V** | **-400 V** |
|---|---|---|---|---|---|---|
| **As-deposited** | **$E_{corr}$ (V vs Ag/AgCl)** | -0.233 ±0.017 | -0.224 ±0.005 | -0.276 ±0.020 | -0.323 ±0.013 | -0.259 ±0.036 |
| | **$i_{corr}$ (nA/cm$^2$)** | 97 ±8 | 221 ±97 | 826 ±120 | 424 ±38 | 184 ±94 |
| | **$i_{pass}$ (μA/cm$^2$)** | 16 ±3 | 16 ±7 | 55 ±10 | 82 ±30 | 62 ±2 |
| **Anodized** | **$E_{corr}$ (V vs Ag/AgCl)** | -0.136 ±0.06 | -0.153 ±0.028 | -0.148 ±0.012 | -0.167 ±0.041 | -0.210 ±0.07 |
| | **$i_{corr}$ (nA/cm$^2$)** | 102 ±58 | 149 ±77 | 105 ±49 | 120 ±28 | 661 ±24 |
| | **$i_{pass}$** | 7.5 ±6.8 | 6.6 ±4.5 | 6.8 ±4.7 | 12 ±2.7 | 47 ±19 |

## References


1. Tang, Y., Liu, Y., Yu, S., Guo, W., Mu, S., Wang, H., Zhao, Y., Hou, L., Fan, Y., and Gao, F. (2015) Template-free hydrothermal synthesis of nickel cobalt hydroxide nanoflowers with high performance for asymmetric supercapacitor. *Electrochim. Acta*, **161**, 279–289.

2. Fan, X., Ohlckers, P., and Chen, X. (2020) One-step and morphology-controlled synthesis of Ni-Co binary hydroxide on nickel foam for high-performance supercapacitors. *Appl. Sci.*, **10** (11).

3. Liu, Z., Ma, R., Osada, M., Takada, K., and Sasaki, T. (2005) Selective and controlled synthesis of α- and β-cobalt hydroxides in highly developed hexagonal platelets. *J. Am. Chem. Soc.*, **127** (40), 13869–13874.

4. Priyadharsini, C.I., Marimuthu, G., Pazhanivel, T., Anbarasan, P.M., Aroulmoji, V., Siva, V., and Mohana, L. (2020) Sol–Gel synthesis of Co3O4 nanoparticles as an electrode material for supercapacitor applications. *J. Sol-Gel Sci. Technol.*, **96** (2), 416–422.
